# *Bacillus subtilis* biofilm development in the presence of soil clay minerals and iron oxides

**DOI:** 10.1038/s41522-017-0013-6

**Published:** 2017-02-09

**Authors:** Wenting Ma, Donghai Peng, Sharon L. Walker, Bin Cao, Chun-Hui Gao, Qiaoyun Huang, Peng Cai

**Affiliations:** 1 State Key Laboratory of Agricultural Microbiology, College of Resources and Environment, Huazhong Agricultural University, Wuhan, 430070 China; 2 Department of Chemical and Environmental Engineering, University of California, Riverside, CA 92521 USA; 3 School of Civil and Environmental Engineering, Nanyang Technological University, 50 Nanyang Avenue, 639798 Singapore

## Abstract

Clay minerals and metal oxides, as important parts of the soil matrix, play crucial roles in the development of microbial communities. However, the mechanism underlying such a process, particularly on the formation of soil biofilm, remains poorly understood. Here, we investigated the effects of montmorillonite, kaolinite, and goethite on the biofilm formation of the representative soil bacteria *Bacillus subtilis*. The bacterial biofilm formation in goethite was found to be impaired in the initial 24 h but burst at 48 h in the liquid–air interface. Confocal laser scanning microscopy showed that the biofilm biomass in goethite was 3–16 times that of the control, montmorillonite, and kaolinite at 48 h. Live/Dead staining showed that cells had the highest death rate of 60% after 4 h of contact with goethite, followed by kaolinite and montmorillonite. Atomic force microscopy showed that the interaction between goethite and bacteria may injure bacterial cells by puncturing cell wall, leading to the swarming of bacteria toward the liquid–air interface. Additionally, the expressions of *abrB* and *sinR*, key players in regulating the biofilm formation, were upregulated at 24 h and downregulated at 48 h in goethite, indicating the initial adaptation of the cells to minerals. A model was proposed to describe the effects of goethite on the biofilm formation. Our findings may facilitate a better understanding of the roles of soil clays in biofilm development and the manipulation of bacterial compositions through controlling the biofilm in soils.

## Soil: Mineral effects on biofilms

The effect of three soil minerals on biofilm production is clarified by research using the common soil bacterium *Bacillus subtilis*. The mineral composition of soil is known to affect biofilm production, but the mechanisms underpinning minerals’ influences have not been well studied. Peng Cai and colleagues at Huazhong Agricultural University in China, with co-workers in the United States and Singapore, studied *Bacillus subtilis* growing in the presence of the minerals montmorillonite, kaolinite, and goethite. Their results suggest the minerals, especially goethite, can encourage biofilm formation by promoting the bursting of bacterial cells. The effect of goethite was attributed to the size of its grains being generally smaller than the bacterial cells. By quantifying the effect of these minerals, the research will assist understanding of biofilm formation and the growth and persistence of bacterial populations in soils.

## Introduction

Microbial biofilms, sessile microbial communities enveloped in a self-produced extracellular polymeric substance (EPS), are ubiquitous in the natural environment.^1^ In soil systems, the sessile state dominates in microorganisms and protects them physically, chemically, and biologically.^2,3^ Soil microbial biofilms play a vital role in the formation and stability of soil aggregates,^4^ weathering of minerals,^5^ and degradation and sequestration of organic carbon.^6^ Therefore, there is a critical need to understand the progressive formation of microbial biofilms and their functions in soil.

The development of microbial biofilms involves five consecutive steps: (1) reversible attachment, (2) irreversible attachment, (3) microcolony formation, (4) biofilm maturation, and (5) dispersion.^7^ In the past decades, extensive studies have been performed on the initial stage of biofilm formation, especially on the adhesion of bacteria on well-characterized minerals and inert surfaces (e.g., kaolinite, montmorillonite, goethite, hematite, quartz, corundum, and glass). The investigated factors include bacterial strain,^8,9^ mineral type,^10,11^ solution chemistry,^12,13^ cell surface features (e.g., proteins and polysaccharides),^14,15^ and organic matter.^16,17^ In the subsequent stage of adhesion, bacteria began to respond to the surface and undergo biological changes in order to overcome the potential energy barrier imposed by the physical–chemical forces, resulting in biofilm formation and maintenance.^18^ For instance, Friedlander *et al.* ^19^ reported that flagella expressed by *Escherichia coli* could reach into surface crevices and act as structural elements in attachment and biofilm formation. A study on titanium plates surfaces treated with anodic oxidation (AO-Ti), alkali-heat (AH-Ti), and acid-alkali (AA-Ti) demonstrated that anodic oxidation treatment was unfavorable for *Staphylococcus aureus* and *E. coli* biofilm formation because the titania coating formed by anodizing had higher antimicrobial property than the other surfaces.^20^ At the initial stage of biofilm formation of *E. coli* K12, single-walled carbon nanotubes came into contact with bacterial cells prior to biofilm maturation and inhibited their growth, but bacteria in mature biofilm were less sensitive to the presence of single-walled carbon nanotubes.^21^ However, the effect of the physical surface–bacterial interactions on the physiological state of a recently attached cell remains poorly understood.

Clay minerals and metal oxides are essential parts of the soil matrix and strongly influence the structure of microbial communities and the formation of biogeochemical interfaces in soils.^22,23^ For example, Steinbach *et al.* ^24^ reported that metal oxides have significant impacts on alkane-degrading community structure and the effects increased during soil maturation. Recently, several studies have examined the effects of soil minerals on the viability of bacterial cells. For example, Wu *et al.* ^25^ found that montmorillonite remarkably improved the metabolic activity of *Pseudomonas putida*, whereas kaolinite and goethite significantly inhibited the activity. Cai *et al.* ^26^ showed that during the examined period of 6 h, *E. coli* O157:H7 retained its viability when attached to montmorillonite and kaolinite, while interaction with goethite was detrimental. Asadishad *et al.* ^27^ examined the inactivation rates of three bacterial strains attached to silica, iron oxide, and alumina surfaces and found a high correlation between the amount of C–metal or O–metal bonds and the extent of bacterial inactivation. All the aforementioned studies are only focused on the metabolic activity of planktonic bacteria in the presence of minerals in several hours, which cannot represent the real physiological state of bacterial biofilm in the soil environment. To our best knowledge, the effects of soil minerals on bacterial biofilm development have never been explored.

In *Bacillus subtilis*, regulation of transcription is critical to biofilm formation.^28^ The transcriptional regulator Spo0A is central to biofilm initiation.^29,30^ It can be activated by various environmental signals. After that, two parallel pathways of anti-repression, *abrB* and *sinR* pathways, are involved, and both of them control, directly or indirectly, the 15-gene *eps* operon that is required for biosynthesis of the extracellular polysaccharides, the *tapA-sipW-tasA* operon,^31^ the *bslA* gene that encodes a hydrophobic biofilm coat protein, and motility genes such as *hag*, *lytA*, and *lytF*.^32–34^ Generally, *abrB* and *sinR* are critical for biofilm formation and can be used to represent cell state.^28^

The main objective of the present work is to study the effects of soil clays (montmorillonite, kaolinite, and goethite) on the biofilm development of the bacterium (*B. subtilis*). Effects of minerals on bacterial biofilm formation and the mechanisms were analyzed by the crystal violet staining method combined with chemical analysis, quantitative real-time polymerase chain reaction (qRT-PCR), confocal laser scanning microscopy (CLSM) and atomic force microscopy (AFM). An enhanced understanding of the relationship between soil clay types and biofilm development will facilitate the manipulation of biofilm growth in natural soil environments.

## Results

### Montmorillonite, kaolinite, and goethite vary in their effects on *B. subtilis* biofilm formation

**Fig. 1:**
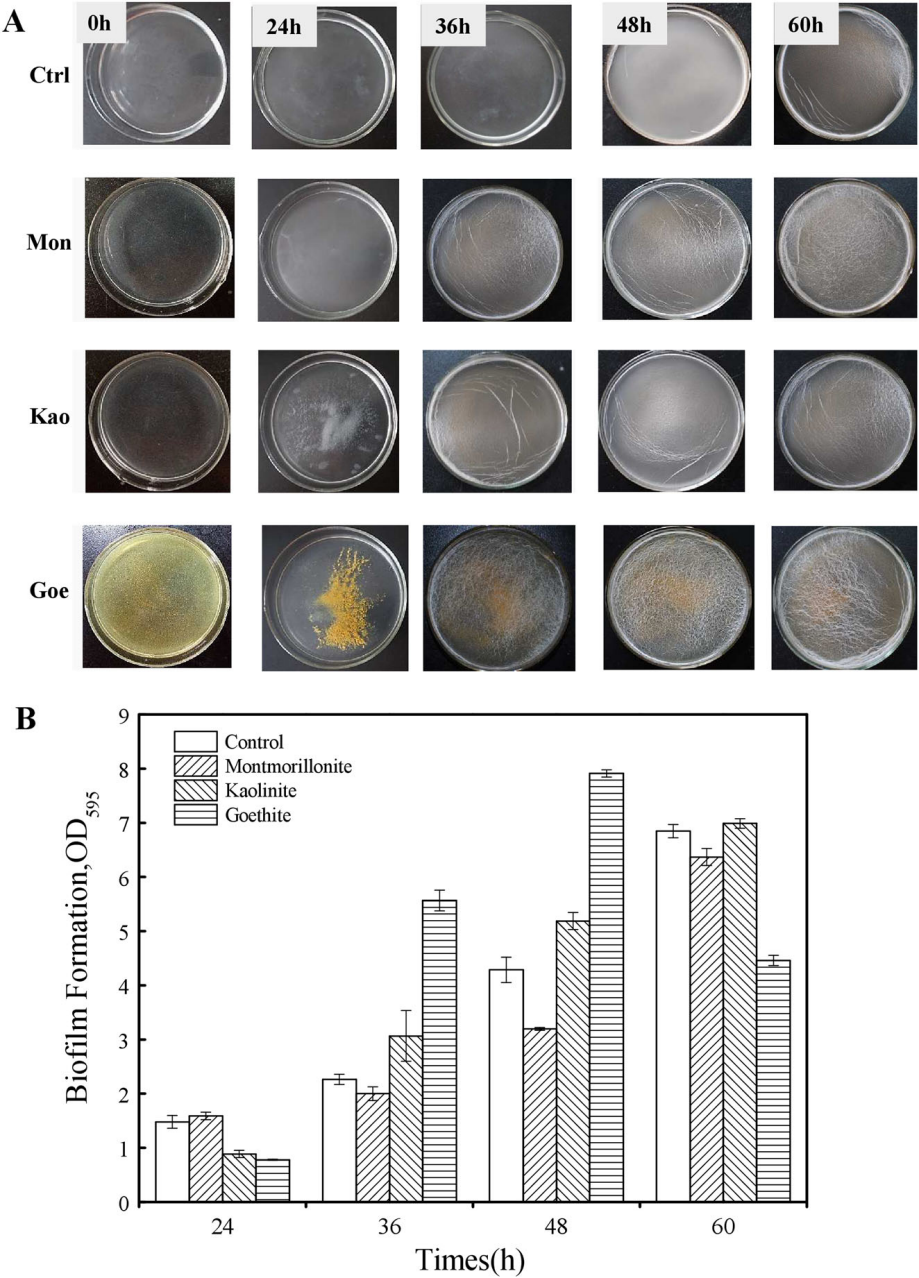
Biofilm formation of *B. subtilis* at differernt time intervals in supplement with different clay minerals. **a** Pellicles with wrinkles were showed for control (Ctrl), montmorillonite (Mon), kaolinite (Kao), and goethite (Goe) at 0, 24, 36, 48, and 60 h as indicated on the left/top, respectively. **b** Quantitive assessment of biofilm formation as determined by crystal violet assays

In order to test the effects of montmorillonite, kaolinite, and goethite on *B. subtilis* biofilm formation, static culture supplemented with 1 g L^−1^ of different minerals was first carried out separately. As shown in Fig. 1a, all the biofilms were formed at the interface of liquid and air, as a pellicle with wrinkles. When compared with the control, the culture with either montmorillonite or kaolinite from 24 to 60 h showed a similar pattern with the largest amount of wrinkles at 60 h. However, the culture supplemented with goethite produced the largest amount of wrinkles at 48 h, followed by a decrease at 60 h (Fig. 1a). These results indicated that these minerals may have different effects on biofilm formation, as wrinkled morphology is a distinctive phenotype in mature biofilms produced by *B. subtilis*.^35^

To quantify the biofilm formation in the presence of different minerals, crystal violet assay and CLSM were performed. As shown in Fig. 1b, the OD_595_ values representing the amount of biofilm in control, montmorillonite, kaolinite, and goethite were 0.49 ± 0.12, 0.53 ± 0.07, 0.30 ± 0.07, and 0.26 ± 0.01 at 24 h, respectively. The biofilm formation of both kaolinite and goethite was impaired at the early stage of biofilm formation, when compared with the control or montmorillonite. With the maturation of biofilm, goethite exhibited the highest biofilm biomass at 48 h, followed by kaolinite, control, and montmorillonite (Fig. 1b). The maximum OD_595_ value of biofilm (2.67 ± 0.63) occurred at 48 h for the goethite suspension culture, which was significantly higher (*p* < 0.05) than that in the control, montmorillonite, or kaolinite systems. From 48 to 60 h, the biofilm biomass of control, montmorillonite, and kaolinite increased continuously, while that of goethite decreased, suggesting its entry into the dispersal stage of biofilm. These results indicated that the bacteria in goethite suspension had a higher biofilm formation speed and capacity than those in the other three systems.

By using the CLSM, the biomass, thickness, and roughness of biofilm were measured at 48 h. Specifically, the total biomass for *B. subtilis* in goethite suspension was 0.67 ± 0.01, which was 3–16 times that of the control, montmorillonite, or kaolinite (Table 1 and Fig. S1). The average and maximum thickness values of the biofilms in goethite were 0.65 ± 0.07 and 6 ± 0.23 μm, respectively, which were significantly higher than those of the other systems (Table 1). The roughness indexes of the biofilm in the control, montmorillonite, kaolinite, and goethite were similar, suggesting that biofilm structure was not affected by the presence of minerals (Table 1). Together, microscopic analysis indicated that, at the earlier stage (48 h), *B. subtilis* biofilm in the goethite suspension was structured, with several layers of largely live bacteria encased within a dense matrix.

**Table 1:** Biofilm structural parameters and biomass content at 48 h

	Control	Montmorillonite	Kaolinite	Goethite
Biovolume (μm^3^/μm^2^)	0.25 ± 0.02	0.09 ± 0.01	0.04 ± 0.007	0.67 ± 0.01
Average thickness (μm)	0.48 ± 0.03	0.09 ± 0.008	0.04 ± 0.002	0.65 ± 0.07
Max thickness (μm)	3.64 ± 0.62	4.16 ± 0.45	2.60 ± 0.17	6.00 ± 0.23
Roughness	1.67 ± 0.025	1.89 ± 0.033	1.94 ± 0.046	1.95 ± 0.51

**Fig. 2:**
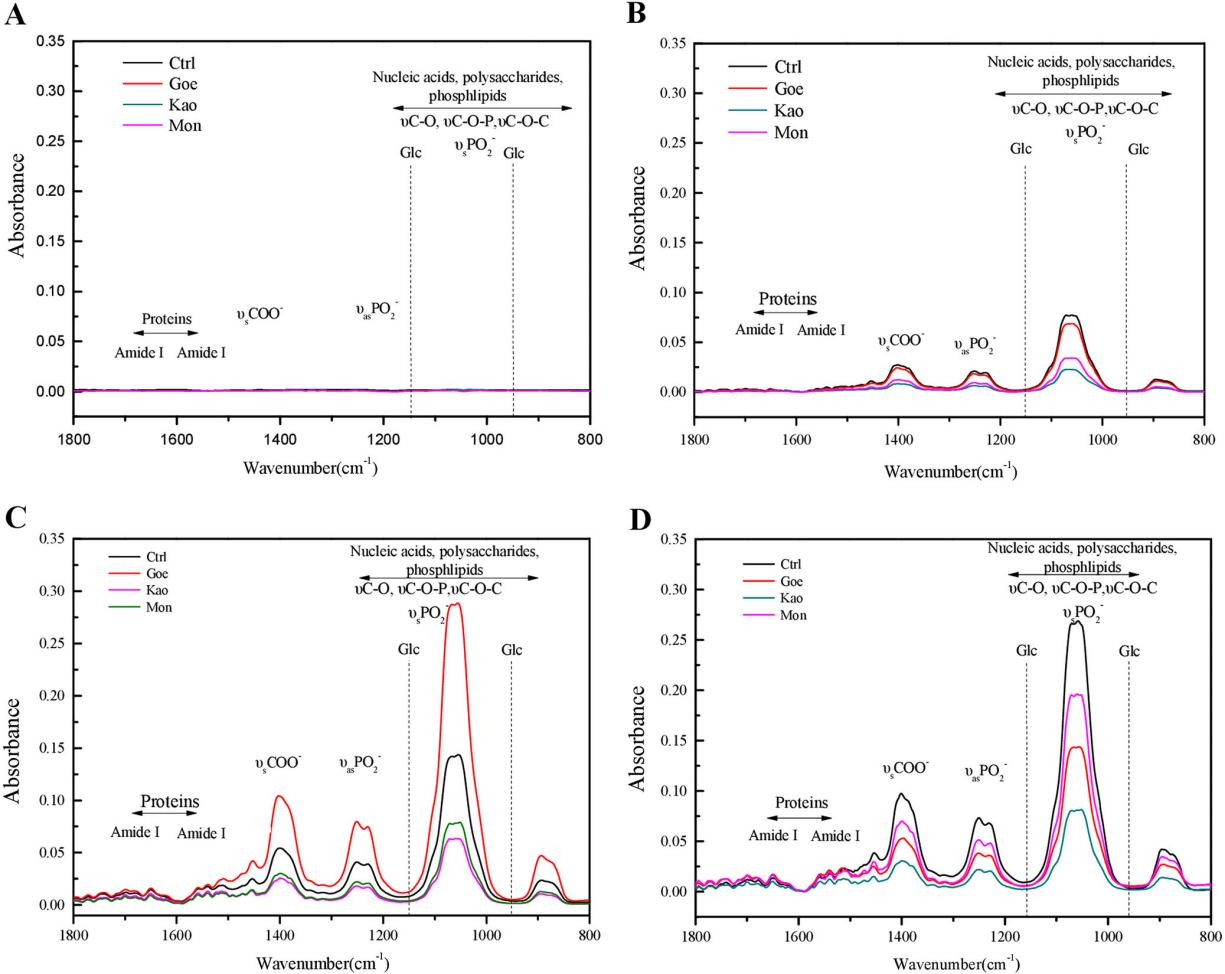
ATR-FTIR assays of the biofilm formation at 24 (**a**), 36 (**b**), 48 (**c**), and 60 h (**d**). At each time point, the absorbance spectra were given for control (Ctrl), goethite (Goe), montmorillonite (Mon), and kaolinite (Kao) seperatedly, as indicated in figure legends. Representive peaks (nucleic acids, polysaccharides, proteins, etc.) were labeled on the top of curves, respectively

In conjunction with the rough surface topography, the *B. subtilis* matrix, which is primarily composed of exopolysaccharides and proteins, provides the biofilm with a remarkably hydrophobic surface that is largely impermeable to aqueous liquids and organic solvents.^32^ Therefore, ATR-FTIR spectroscopy analysis was performed to check whether exposure to different minerals induces changes in the matrix components during *B. subtilis* biofilm formation. The results showed that the shape and peaks of wave number of IR bands did not change notably among all samples (Fig. 2, and Table S2), suggesting that the components of biofilm were not affected by different minerals although the biofilm formation and dispersal varied from each other. Furthermore, the amount of exopolysaccharides and proteins in *B. subtilis* biofilm matrix during biofilm formation was detected by the chemical analysis and the results are shown in Table S3. The production of EPSs by *B. subtilis* started at the onset of biofilm formation. Similar trends for the release of exopolysaccharides in biofilm were observed among the control, montmorillonite, and kaolinite systems. The amount of exopolysaccharides reached the peak at 60 h, but that in the presence of goethite reached the maximum of 11.79 ± 0.10 mg g^−1^ at 48 h, followed by a decrease with biofilm dispersal. The proteins in the biofilms of the control, montmorillonite, and kaolinite all reached the maximum at 60 h, while those in goethite suspension reached the peak at 48 h. The changes of exopolysaccharides and proteins in biofilm were consistent with the variation of biofilm formation.

### Mineral-induced cell death contributes to enhanced biofilm formation

**Fig. 3:**
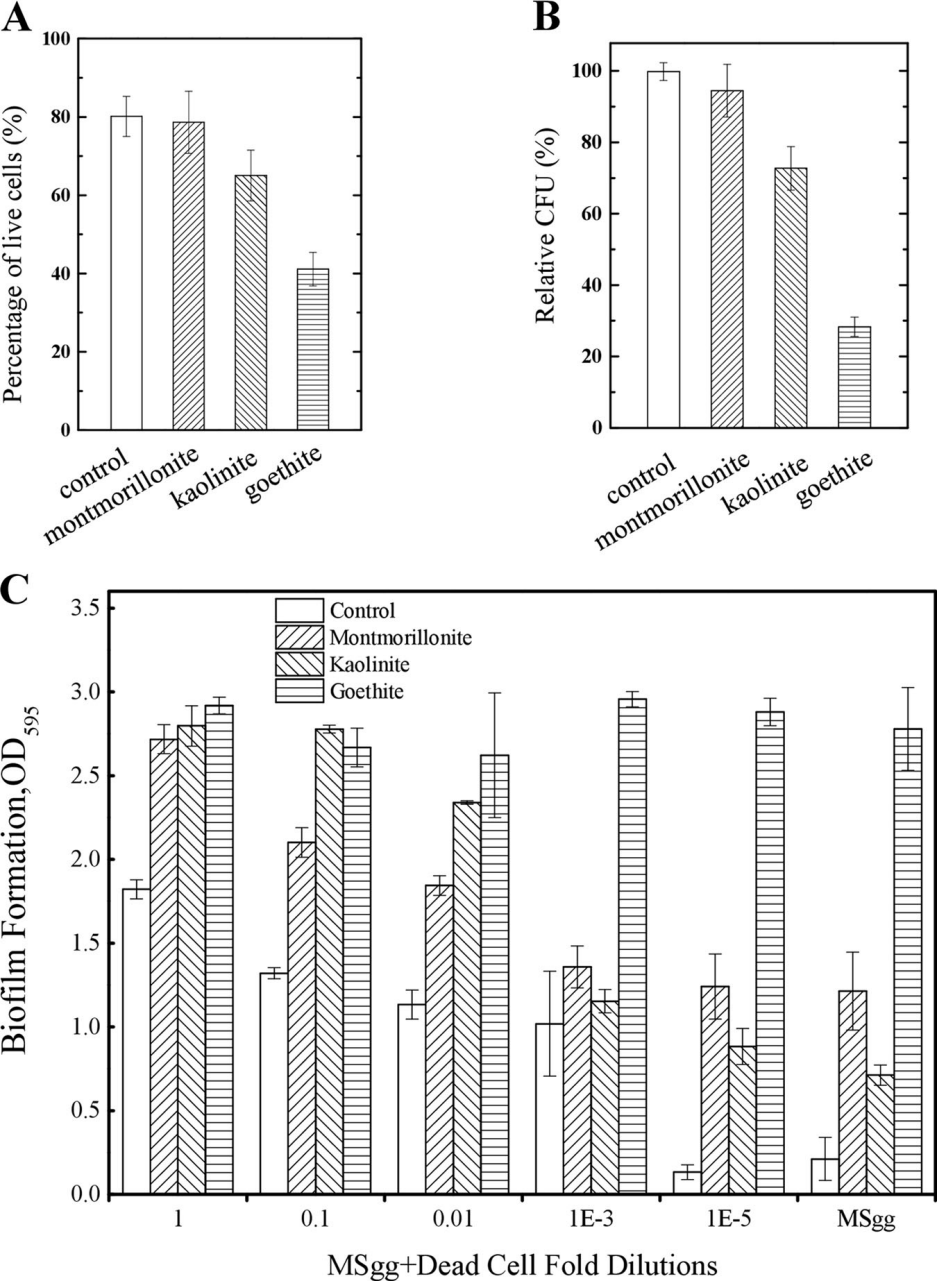
Mineral-induced lethal effects and their role in biofilm formation. **a** Live/Dead staining assays. Except for the control, cells were exposed to minerals for 24 h and their viabilities were determined and given as percentages. **b** CFU of control and mineral-exposed cells. CFUs were normalized to the control and showed as relative percentages, respectively. **c** Effects of different concentrations of dead cells on *B. subtilis* biofilm formation under different treatments

The effects of mineral contact on cell viability were studied by different assays. First, Live/Dead fluorescent staining assays were performed with mineral-coated glasses after 24 h exposure as described in methods. Cell viabilities for bacteria on both glass surface and montmorillonite-coated surface were around 80%, with a slight decrease on kaolinite-coated surface (65%), and the lowest for cells attached to goethite (40%) (Fig. 3a and Fig. S1). Overall, the cell viability was the lowest for goethite, followed by kaolinite and montmorillonite. The viability between montmorillonite and control was comparable, indicating that the former may have no killing effect on cells. However, the other two minerals, kaolinite and goethite, could induce cell death in different extents. The pattern of mineral-induced cell death was similar to that of biofilm biomass at 24 h (Fig. 1).

In addition, cell viabilities in broth cultures with mineral suspensions were quantified by colony-forming unit ﻿(CFU) assays. As shown in Fig. 3b, similar results were obtained, with the lowest viability for goethite, followed by kaolinite, montmorillonite, and the control, with the control defined as 100% of viability in the assay. The results indicated that mineral exposure will cause cell death, which is probably the main reason for the changes in biofilm formation when exposed to different minerals.

To confirm this predication, cultures supplemented with different concentrations of dead cells were performed as described in methods. As shown in Fig. 3c, dead cells were fold-diluted and added into broth cultures, followed by measuring the biofilm formation at 48 h. Except for goethite, all the biofilm biomasses of the other cultures were increased, more or less, when dead cells were added, indicating that the more the dead cells, the higher the biofilm biomass (Fig. 3c). The results support our previous hypothesis that the dead or damaged cells may be the main reason for enhanced biofilm formation in goethite, which can serve as nutrients for living cells.

### The bacteria sense and response to mineral exposure

**Fig. 4:**
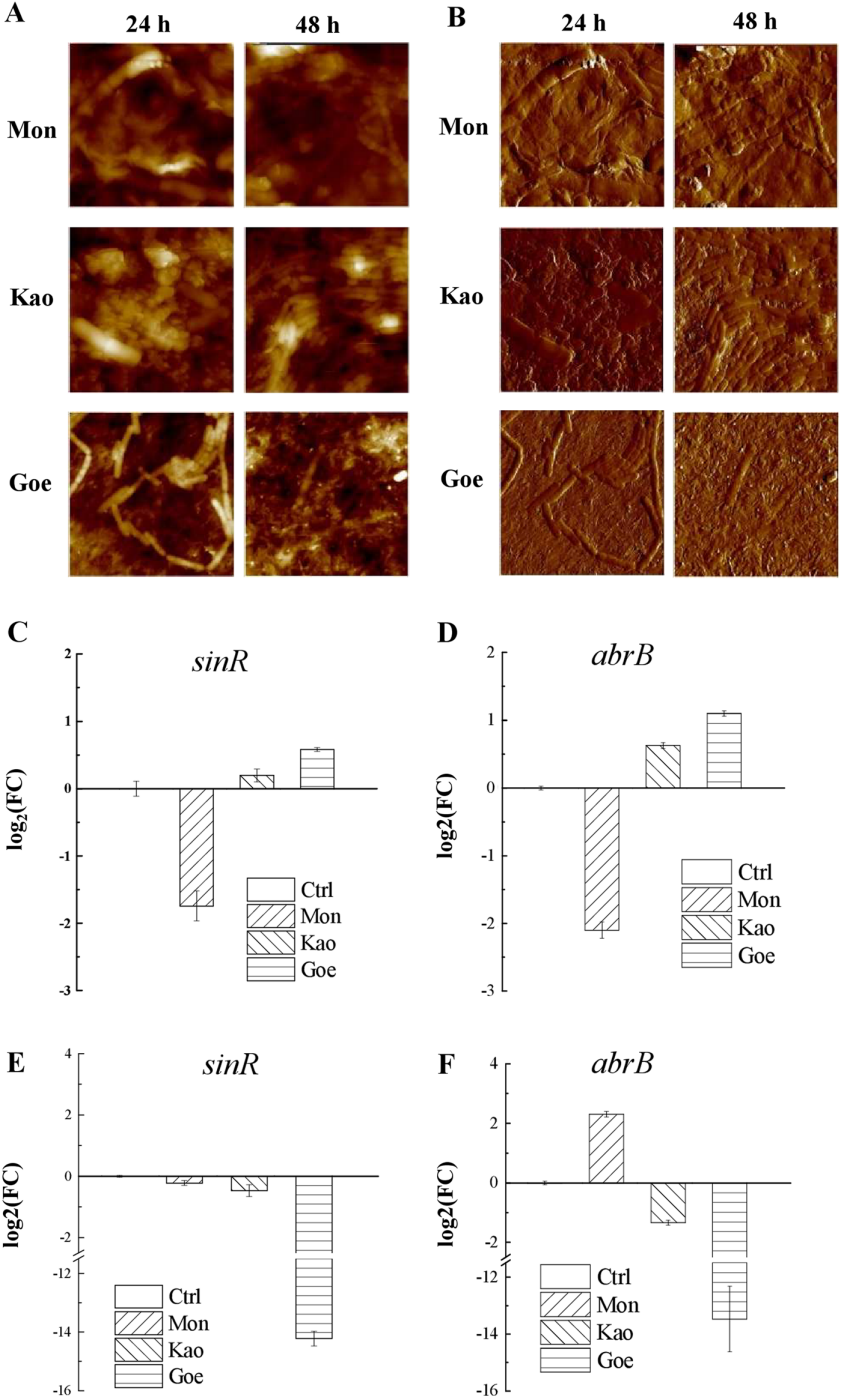
Contact of minerals and bacteria cells, and the resulting gene experssion changes. AFM height (**a**) and peak force images (**b**) of *B. subtilis* biofilm formed on mineral surfaces after 24 and 48 h (20 × 20 μm^2^). *Top panel*: *B. subtilis* on montmorillonite-coated surface (Mon); *Middle panel*: *B. subtilis* on kaolinite-coated surface (Kao); *Bottom panel*: *B. subtilis* on goethite-coated surface (Goe). **c**–**f**: Quantitative expression analyses of the *sinR* (**c**, **e** for 24, 48 h, respectively) and *abrB* (**d**, **f** for 24, 48 h, respectively) genes. Both of the genes are critical regulators that involved in the regulation of *B. subtilis* biofilm formation and cell motility

The interface of bacteria and mineral surfaces was studied by atomic force microscopy (AFM) analysis (Fig. 4a, b). Chain-like structures of bacterial cells are formed on minerals-coated surfaces at 24 h. However, only a few single cells were found on the goethite-coated surface at 48 h. The interaction between goethite and bacteria is so strong that cell wall was punctured and may injure bacterial cells (Fig. 4a, b, bottom panel). In broth cultures, minerals tend to deposit on the bottom of the flask and biofilm is formed on the liquid surfaces (Fig. 1). Therefore, the AFM results indicated that bacteria cells are sensitive and responsive to minerals and try to stay away from goethite, by swimming to the air–liquid interface.

To evaluate the bacterial states, qRT-PCR assays were performed to measure the expression of two transcriptional regulatory genes, *sinR* and *abrB*. Since the two genes play central roles in regulating the *Bacillus* biofilm formation, the expression of *sinR* and *abrB* can explain cell mobility and cell differentiation. As shown in Fig. 4c, the expression of *sinR* was slightly upregulated in kaolinite and goethite, and downregulated in montmorillonite at the early stage of biofilm formation (24 h), suggesting the preference of cells in a planktonic state. The expression of *sinR* was significantly downregulated at 48 h in goethite (Fig. 4e), suggesting that the mobility of cells was reduced and EPS formation was enhanced. Similarly, the expression of *abrB* in goethite/kaolinite was upregulated at 24 h (Fig. 4d) and downregulated at 48 h (Fig. 4f).

**Fig. 5:**
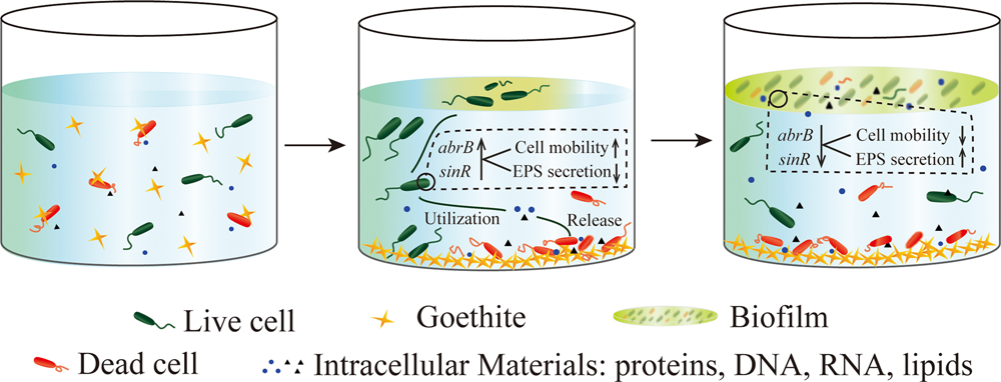
Proposed model for the effects of goethite on *B. subtilis* biofilm formation. Bacteria interact with goethite in suspension and the adsorption to goethite of bacteria can induce cell damage or death (*left panel*). Live cells utilize the intracellular materials released by damaged or dead cells. In response to lethal stress, the *abrB* and *sinR* genes, which are the key to the transformation of bacilli between biofilm and free-living mode-of-life, are upregulated and this increase cell mobility and inhibit EPS secretion. In order to avoid damage of goethite, bacteria prefer to move to air–liquid interface (*middle panel*). As bacteria aggregate on the air–liquid interface, both *abrB* and *sinR* are downregulated, which decreases cell mobility and increases EPS secretion, and the biofilm is formed rapidly (*right panel*)

Interestingly, qRT-PCR results showed that the biofilm formation in goethite was not enhanced but suppressed during the early stage (24 h), despite a faster biofilm formation than that of the others (Fig. 1). However, this is reasonable and consistent with the results of biofilm quantification (Fig. 1). In our consideration, the bacteria cells need to maintain their motilities and to make it possible for them to keep away from lethal materials. On the basis of our research, we proposed a model to describe the effect of goethite on biofilm formation. As shown in Fig. 5, when goethite is added, cells may adhere to mineral particles leading to cell death. Dead cells then precipitate with goethite, and cell components, including nucleic acids, proteins, and/or signal molecules, are released. Living cells are sensitive and responsive to the lethal effect, and aggregate on the surface of culture. Along with cell aggregation and EPS secretion from the bacteria, biofilm is formed quickly.

## Discussion

Various microorganisms are distributed in the soil and they usually form multispecies biofilm.^36^ However, the interactions between soil components and microorganisms remain poorly understood. In the current study, the interaction between three common soil minerals (montmorillonite, kaolinite, and goethite) and a typical soil bacterium, *B. subtilis*, was studied. We found that the minerals vary in their effects on bacterial biofilm formation, and mineral-induced cell lysis is the major contribution that accelerate biofilm formation with the corresponding mineral. Furthermore, a model was proposed to describe the process of biofilm formation under mineral exposure.

Generally, the biofilm formation of *B. subtilis* can be divided into three stages: the early stage, maturation stage, and dispersal stage.^3^ Due to exposure to different minerals, the time interval for each stage may be different. As shown in Fig. 1, the maturation of biofilm in goethite reached the peak at 48 h, while that of the control, montmorillonite, or kaolinite reached the maximum at 60 h, at which time the biofilm biomass of goethite was already on the decline. The maximum biofilm biomass was higher in goethite than in the other three systems, but the addition of more dead cells in goethite showed no effects on biofilm formation, suggesting the existence of ceiling effect (Fig. 4). Under the exposure to goethite or other lethal minerals, the biofilm formation process will be accelerated. Similar results could also be found in other study in which the biofilm formation of *E. coil* K12 was enhanced after 48 h of exposure to single-wall carbon nanotubes in the concentration range of 5–300 mg L^−1^.^21^

Physical attributes and mineral chemistry are believed to be main intrinsic factors that explain how minerals characteristics influence bacterial colonization.^37^ The equivalent diameters of montmorillonite, kaolinite, and goethite were 1.89 ± 0.36, 1.57 ± 0.17, and 0.20 ± 0.10, respectively.^38^ As compared with that of *B. subtilis*, the size of first two minerals were larger than that of bacteria cells, while that of goethite was smaller than that of cells. These differences of size resulted in different surface morphology of minerals, where goethite surface is needle-shaped and montmorillonite surface is flat (Fig. 4).^26,39^ In addition, the surface hydrophobicity of minerals was different. The contact angles between H_2_O to montmorillonite, kaolinite, and goethite were 50.6 ± 2.8, 39.2 ± 1.2, and 21.6 ± 1.1, respectively,^38^ suggesting that hydrophobicity of goethite is the least. Furthermore, the surface of goethite is positively charged, and that of montmorillonite and kaolinite are negatively charged.^26,38^ Deposition of *E. coli* on goethite is the greatest, following by kaolinite and montmorillonite.^26^ Notably, mineral composition, or (more accurately) metal ion which may be dissolved from mineral, is believed to have no significant impact to *B. subtilis* biofilm formation. One reason is that the solubility of these minerals is very low in water. And the other one reason is that the medium (Msgg) used to cultivate *B. subtilis* has plenty of metal ions (2 mM MgCl_2_, 700 μM CaCl_2_, 50 μM MnCl_2_, 50 μM FeCl_3_, 1 μM ZnCl_2_). As a conclusion, the variances of both physical (e.g., morphology, roughness) and chemical (e.g., surface charge) properties of minerals are attributed to different effects of mineral to *B. subtilis* growth and biofilm formation.

In a previous study, we reported that there was no energy barrier between *B. subtilis* and goethite, and the energy barrier of *B. subtilis* with kaolinite and montmorillonite was 48.81 and 23.80 kBT, respectively, indicating that goethite has an intense interaction with *B. subtilis* cells, which may injure and even kill the cells.^38,39^ The goethite is a kind of nanoparticle mineral and the goethite-coated surface may be unsuitable for cell growth (Figs. 3, 4). Dead cells may be utilized as nutrients by the living cells, promoting biofilm formation (Fig. 3c). Among the three minerals, goethite is the most detrimental, montmorillonite is not as detrimental as goethite to *B. subtilis*, and kaolinite is in-between (Fig. 3a, b). In broth culture, cell with kaolinite has similar sediment as goethite did (Fig. 1a). So did the accumulation of biofilm biomass (Fig. 1b) and gene-expression profiles (Fig. 4c–f). As a result, the biofilm formation with kaolinite is between that with montmorillonite and goethite and more similar to goethite. It seems that bacterial cells could not distinguish from different minerals, or the enhancement of biofilm formation by mineral exposure is not specific. The proposed model can be reliably used to interpret other minerals.

Mineral contact or exposure is a kind of stress condition to *B. subtilis*, and the variances between different minerals lead to different levels of cell responses. As been noted by Uroz *et al*.,^37^ mineral is likely to regulate gene expression in a certain extent. *B. subtilis* is a model organism and the regulatory mechanism to cell states, which is either planktonic free-living or biofilm sessile state, is well documented,^28^ therefore we can distinguish cell state by determine the gene expression of the central regulators *abrB* and *sinR*. Generally speaking, when *Bacillus* cells are going to form biofilm, both *abrB* and *sinR* are upregulated, thus matrix genes (*eps*, *tapA*, *bslA*) are turned off and motility genes (*hag*, *lytA*, *lytF*) are turned on. In contrary, when *abrB* and *sinR* are downregulated, matrix genes are turned on and motility genes are turned off, and cells are likely to be in the planktonic free-living state. *B. subtilis* is a highly motile bacteria specie.^40,41^ Turning on motility genes at early stage is beneficial to *B. subtilis* to get away from detrimental minerals (Fig. 4c, d). Likewise, turning off motility genes and turning on matrix genes will enhance biofilm formation, particularly that for goethite both *abrB* and *sinR* are downregulated more than 14-fold and 12-fold, respectively (Fig. 4e, f). Since BslA is a bacterial hydrophobin that coats *B. subtilis* biofilm, the biofilm is on the liquid–air interface, and the culture broth is becoming very clear during biofilm maturation (Fig. 1a).

The mineralosphere, which has been defined as the specific interface and habitat encompassing the rocks and the surrounded soil, is under the influence of minerals.^37^ The results from the current study provide useful information on how mineralosphere is formed. By using qRT-PCR, we showed that mineral contact resulted in significant changes in gene expression and biofilm formation (Fig. 4c–f). Along with biofilm formation, bacterial surface properties, including adsorbability, hydrophobicity, and nutrient capture, underwent change accordingly, and the changes could be enlarged in the subsequent processes.

## Materials and methods

### Bacterial strains and culture conditions

Laboratory isolates of *B. subtilis* were obtained from the State Key Laboratory of Agricultural Microbiology, Huazhong Agriculture University (Wuhan, China). Medium used throughout this study was Minimal medium (MSgg) (5 mM potassium phosphate buffer pH 7, 100 mM Mops pH 7, 2 mM MgCl_2_, 700 μM CaCl_2_, 50 μM MnCl_2_, 50 μM FeCl_3_, 1 μM ZnCl_2_, 2 μM thiamine, 0.5% glycerol, 0.5% glutamate, 50 μg mL^−1^ tryptophan, 50 μg mL^−1^ phenylalanine).^32^ Colonies of *B. subtilis* were initially inoculated into 10 mL of MSgg, and incubated at 28 °C, 180 rpm. Then 1 mL culture was transferred to a volume of 100 mL of MSgg, and the bacteria were incubated for 22 h at 28 °C, 180 rpm. All cultures were harvested at the late-exponential phase by centrifugation for 30 min at 4500 g, followed by two washings with sterilized distilled-deionized water (ddH_2_O) to remove any trace of the growth medium. The bacterial concentration was adjusted to an optical density of 0.5 at 600 nm (OD_600_), corresponding to approximately 10^8^ CFU mL^−1^. The correlation between the optical density and viable cell count was determined by plate counts on LB agar.

### Clay minerals and iron oxide

Kaolinite and montmorillonite were purchased from the Clay Minerals Society (USA). Goethite was synthesized by the simultaneous addition of a 0.15 M Fe(NO_3_)_3_ solution with the neutralizing 2.5 M KOH in a high-density polyethylene bottle as previously reported.^26^ Mineral suspensions with a concentration of 1 g L^−1^ were prepared using ddH_2_O. The clay mineral suspensions and goethite suspension were disaggregated using a Sonic Dismembrator (Branson Sonifier 450) for 30 min at ~160 W and then autoclaved for 30 min at 121 °C.

### Assays of biofilm development in suspended minerals

A microtiter biofilm was assayed in a semiautomatic microtiter plate assay using a modification of a protocol as previously described by Harmsen *et al.* ^42^ Briefly, a total of 150 μL MSgg and 30 μL mineral suspensions were placed in each well of U-bottom polystyrene 96-well microtiter plates (Costar, Corning Incorporated, Corning, NY), and eight replicate wells were used for each analysis. Then 1.5 μL bacterial suspension was added to each well, followed by incubating plates at 28 °C for 0, 24, 36, 48, and 60 h. A pipetting robot removed the culture, washed the cells twice with 200 μL sterile ddH_2_O, and added 200 μL of a 0.1% (wt/vol) crystal violet solution in ddH_2_O. After 15 min of staining, the crystal violet was removed, followed by two wash cycles performed with 200 μL sterile ddH_2_O, and the addition of 200 μL ethanol. The optical density of the eluted crystal violet was determined at a wavelength 595 nm using a microplate reader (synergy HT, BioTek). The minerals without bacteria served as control. For biofilm formation assay with 96-well plates, there were eight replicate wells for every treatment and four wells for blank control. The analysis was repeated at least five times with similar results.

### CLSM and image analysis

For CLSM observations, biofilms were grown in a polystyrene 6-well microtiter plate (Costar, Corning Incorporated, Corning, NY) as described in the section “Assays of biofilm development in suspended minerals”. After 48 h of development at 28 °C, biofilm was gently picked out and washed with phosphate buffer saline (PBS) to eliminate any free-floating bacteria. Biofilms were fluorescently tagged by adding SYTO9 (Molecular Probes Inc.) following the manufacturer’s instructions. A mixture of stains was prepared in PBS buffer with a final concentration of 2.5 μM for SYTO 9. The biofilm and the mixture were added together, followed by incubation in the dark for 15 min and visualizing the biofilm with an Olympus confocal laser scanning microscope equipped with a 63 × oil immersed objective and a 20 × dry objective (Olympus, Japan). Each confocal image stack was saved as a series of tiff images for analyses in COMSTAT.^43^ In COMSTAT, thresholding of the image is firstly performed by applying a fixed threshold value, following by a segmentation process that removes biomass pixels which are not in some way connected to the substratum.^43^ For each image stack, total biomass (µm^3^/µm^2^), the average and maximum thickness, and the roughness coefficient, as an indicator of biofilm heterogeneity, were calculated by COMSTAT from the images. For COMSTAT analysis, at least five images were chosen randomly for every sample and every analysis was repeated three times.

### Attenuated total reflectance-Fourier transform infrared spectroscopy (ATR-FTIR) experiments

The ATR-FTIR spectra were recorded on a Vertex 70 spectrometer (Bruker Optik GmbH). The biofilms were formed and analyzed at 24, 36, 48, and 60 h. The *B. subtilis* mineral suspensions were prepared by mixing the media, mineral, and bacteria at the ratio of 5:1:0.05. At 24 h when biofilms were not well formed, the suspension was used for test. Biofilms formed at 36, 48, and 60 h were carefully picked out and washed twice with ddH_2_O, followed by removal of redundant liquid. The background spectrum consisting of the combined absorbance of the ZnSe crystal and MSgg was recorded from 64 scans in the 400–4000 cm^−1^ at a 4 cm^−1^ resolution, and all successive spectra were subtracted this background spectrum.

### Effect of clay minerals on bacterial survival

To test the bacterial survival on mineral surfaces, mineral-coated round glass coverslips were prepared as previously described.^39^ Briefly, 0.4 mL of the ultrasound mineral suspensions (1 g L^−1^) were pipetted onto the circle coverslips and the minerals onto the glass substrate were boiled at 120 °C for 20 min. Then deionized water was used to rinse the coverslips and dried at 60 °C. The mineral-coated coverslips were autoclaved for 20 min at 121 °C. Next, the as-treated-coated coverslips were placed in sterile polystyrene 6-well plates (Costar, Corning Incorporated, Corning, NY) and supplemented with 0.25 mL of bacterial suspension. After bacterial adhesion for 30 min, 4.75 mL MSgg was added. The 6-well plates were incubated statically in the dark at 28 °C for 24 h. Finally, the coverslips were rinsed gently at least three times in deionized water and prepared for Live/Dead fluorescent staining assays.

The viability of cells adhered to minerals was evaluated in this study using Live/Dead BacLight Bacterial Viability Kits (Thermo Scientific, USA), which have been widely used to enumerate viable bacteria. With a mixture of the SYTO-9 and propidium iodide stains, bacteria with intact cell membranes were stained fluorescent green (considered to be viable), whereas bacteria with damaged membranes were stained fluorescent red (considered to be dead).

For clay mineral and bacterial interaction, 200 µL clay mineral suspension and 10 µL bacteria with 1 mL MSgg were mixed in a 2 mL sterile centrifuge tube. After shaking at 180 rpm for 4 h, bacteria were then immediately spread on LB agar plates and incubated overnight at 28 °C for CFU enumeration.

### Biofilm formation in the presence of dead cells

Biofilm development in the presence of dead cells at different concentrations was monitored with sterile, non-treated 96-well flat-bottom microtiter plates. The purpose of these experiments was to better interpret the results of biofilm formation in the presence of different minerals. Specifically, these experiments attempted to test the hypothesis that dead cells can serve as a nutrient source to enhance biofilm development. The cells were inactivated by autoclave treatment. To ensure that all the cells were dead, a sterility test was performed in triplicate by plating 50 µL of autoclaved cells on LB plates. A serial dilution (10^0^, 10^−1^, 10^−3^, 10^−5^) of the 3 × 10^7^ CFU mL^−1^ dead cells was performed in MSgg to obtain different concentrations of dead cells for the biofilm assay. The biofilm growth procedure was the same as described above, except that instead of MSgg different concentrations of dead cells were used. The biofilms were measured after 48 h of growth at 28 °C using the same procedures described in the section “Assays of biofilm development in suspended minerals”.

### Morphology measurements of *B. subtilis* biofilm on mineral surfaces using AFM

Generally, the samples were prepared in the same way as described in Live/Dead staining assay. The 6-well plates were statically positioned in a biochemical incubator in the dark at 28 °C for 24 and 48 h. The coverslips were picked out and dried in another clean 6-well plates for 2 h. AFM were carried out using a MultiMode 8 AFM with a NanoScope V controller (Bruker, Germany). Topographical images of the biofilms were performed by the ScanAsyst mode using ScanAsyst-Air cantilevers with 0.4 N m^−1^ nominal spring constant (Bruker, Germany). All images were collected with a resolution of 512 × 512 pixels and a scan rate of 1 Hz.

### Quantitative real-time polymerase chain reaction (qRT-PCR)

qRT-PCR was performed on samples of *B. subtilis* exposed to montmorillonite, kaolinite, and goethite for different time intervals (24 and 48 h). The obtained biofilm cells were disintegrated using liquid nitrogen and kept on ice. Total RNA was extracted and purified with the Trizol (Life Technologies, USA), according to the manufacturer’s instructions. The RNA concentration was calculated by measuring the absorbance at 260 nm and 300 ng of RNA was used for cDNA synthesis using the M-MLV Reverse Transcriptase Kit (BIO-Rad). qRT-PCR was carried out in the ViiA™ 7 Real-Time PCR System (Thermo Scientific, USA) using the Power SYBR Green PCR Master Mix (Applied Biosystems, USA), with the primers listed in Table S1. Conditions for qRT-PCR were as follows: 50 °C for 2 min, initial denaturation at 95 °C for 10 min, and 45 cycles of 15 s at 95 °C and 1 min at 60 °C. To confirm the specificity of the amplification products, melting curve analysis and agarose gel electrophoresis were performed to verify the presence of the targeted amplicons. The threshold cycle method (2^−ΔΔCT^)^44^ was used to analyze changes in gene expression in a given sample relative to a reference sample (which is a control sample of biofilm cells without minerals) and the data were normalized to the reference 16S RNA. For each sample, qRT-PCR was performed in triplicate and the entire experiment was repeated twice with RNA samples extracted from independent cultures.

## Electronic supplementary material

Supplementary Information
