# Supplementary material for: Bacillus subtilis biofilm development in the presence of soil clay minerals and iron oxides
Source: NPJ Biofilms Microbiomes. 2017 Feb 9;3:4. doi: 10.1038/s41522-017-0013-6 (PMC5445608; doi:10.1038/s41522-017-0013-6)
Supplement: Supplementary file 1 — Supplementary Information [file 41522_2017_13_MOESM1_ESM.pdf]

Supplementary Information of

Bacillus subtilis Biofilm Development in the Presence of Soil Clay Minerals and Iron Oxides

Wenting Ma[1], Donghai Peng[1], Sharon L. Walker[2], Bin Cao[3], Chun-Hui Gao[1], Qiaoyun Huang[1], Peng Cai[1]*

1: State Key Laboratory of Agricultural Microbiology, College of Resources and Environment, Huazhong Agricultural University, Wuhan 430070, China

2: Department of Chemical and Environmental Engineering, University of California, Riverside, California 92521, USA

3: School of Civil and Environmental Engineering, Nanyang Technological University, 50 Nanyang Avenue, 639798, Singapore

*Corresponding author: Peng Cai

State Key Laboratory of Agricultural Microbiology

Huazhong Agricultural University, Wuhan, China

Phone: +86 27 87671033; Fax: +86 27 87280670

E-mail address: cp@mail.hzau.edu.cn

Contents

1. Supplementary Methods

1.1 Extracellular Polymeric Substance Isolation and Characterization.

1.2 References

2. Supplementary Tables

Table S1. Primers designed for this study.

Table S2. Identification of absorption bands in the Mid-IR spectra of *B. subtilis* biofilm.

Table S3. The concentration of polysaccharides and proteins in biofilm (mg g^{-1}).

3. Supplementary Figures

1. Supplementary Methods

1.1 Extracellular Polymeric Substance Isolation and Characterization.

Due to their importance in biofilms, the extracellular polymeric substances (EPS) at different stages of biofilm development were extracted and characterized for their sugar and protein content as previously described by Steinberger and Holden.[1] Polysaccharide was determined using the sulfuric acid-phenol method as described by Dubois *et al.*[2] with a glucose standard. Proteins were measured by bicinchoninic acid (BCA) (Boisynthesis Co., Ltd., Beijing).

1.2 References

1. Jiang W, Saxena A, Song B, Ward BB, Beveridge TJ, Myneni SC Elucidation of functional groups on gram-positive and gram-negative bacterial surfaces using infrared spectroscopy. *Langmuir* **20**, 11433-11442 (2004).
2. Ojeda JJ, Romero-González ME, Bachmann RT, Edyvean RG, Banwart SA Characterization of the cell surface and cell wall chemistry of drinking water bacteria by combining XPS, FTIR spectroscopy, modeling, and potentiometric titrations. *Langmuir* **24**, 4032-4040 (2008).

2. Supplementary Tables

Table S1. Primers designed for this study

Primer Name	Direction	Sequence(5’ to 3’)
abrB	F	GGAATCGCAGAAAAAGATGC
	R	TTTCGCTGATGATTTGCTCA
sinR	F	TTGGCCAGCGTATTAAACAA
	R	GAACAGCGGAGACTTTTTCG

Table S2. Identification of absorption bands in the Mid-IR spectra of *B. subtilis* biofilm

Wavenumber (cm^{-1})	IR band assignment
1652-1637	amide I:C=O stretching, -CN and -NH bending in amines
1550-1540	amide II: N-H bending, C-N stretching
1454-1482	bending of CH_2 /CH_3
1360-1450	υ_s (COO^-)
1210-1270	υ(C-OH) in COOH; P=O stretch in phosphates
1150-950	asymmetric and symmetric stretching of phosphate PO_2 and $P(OH)_2$
1048-1078	C-OH, C-O-C, and C-C vibrations of polysaccharides

Table S3. The concentration of polysaccharides and proteins in biofilm (mg g^{-1})

	Control		Montmorillonite		Kaolinite		Goethite	
	Polysaccharides	Proteins	Polysaccharides	Proteins	Polysaccharides	Proteins	Polysaccharides	Proteins
24h	4.39±0.26	4.40±0.87	6.45±0.95	6.94±0.36	5.48±0.62	6.67±0.88	6.12±0.76	7.23±0.61
36h	5.96±1.05	6.42±0.55	5.96±0.74	8.20±0.64	5.94±0.78	8.22±0.66	7.03±0.63	9.83±0.47
48h	6.08±0.32	7.20±0.45	5.91±0.99	10.03±0.71	6.08±1.08	9.61±0.71	11.79±0.10	14.51±0.10
60h	11.75±0.65	11.30±0.96	13.29±1.34	14.77±1.50	12.46±0.35	14.01±1.72	8.88±1.84	13.12±1.22

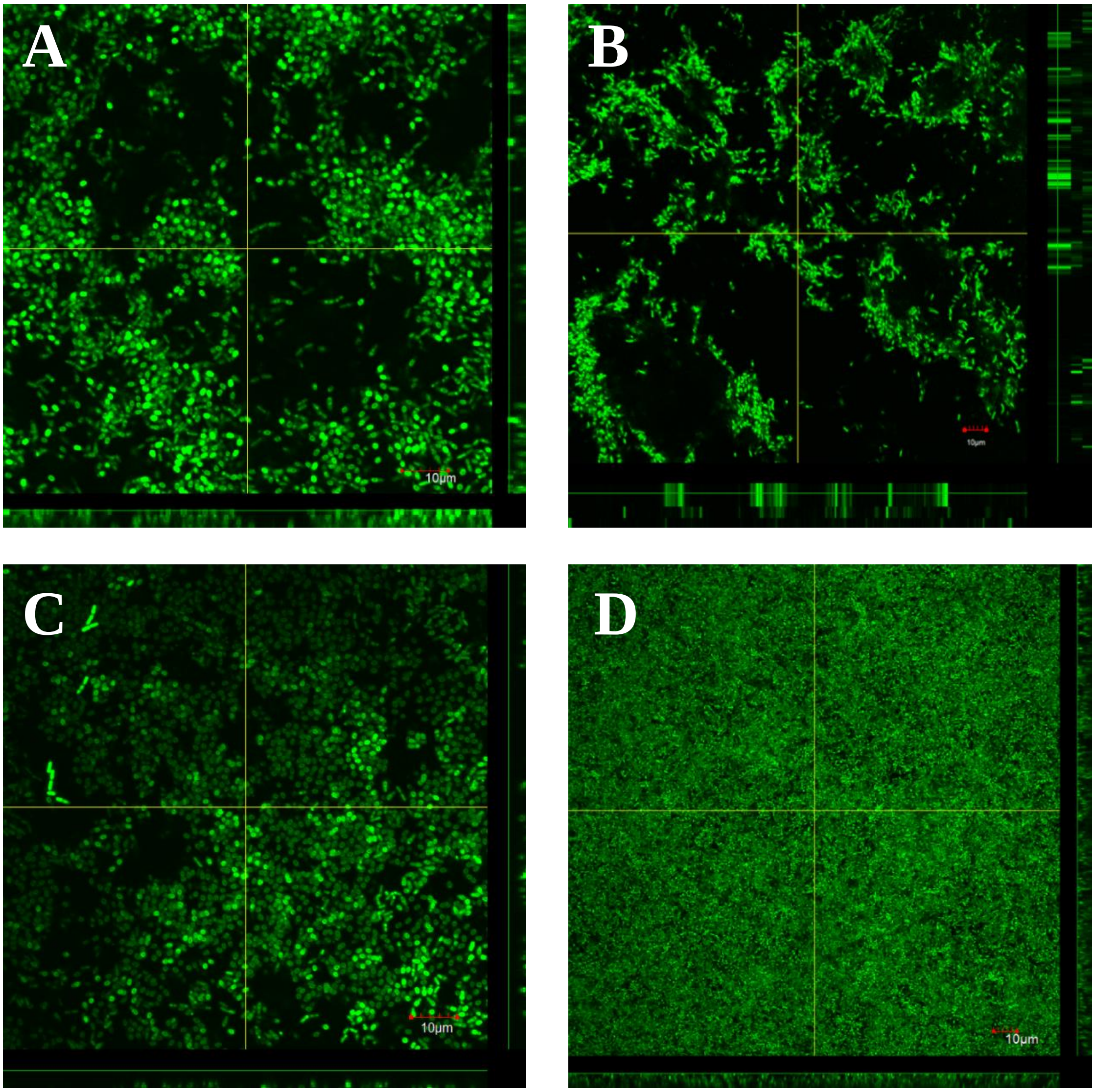

Figure S1. CLSM images of *B. subtilis* biofilm formed in MSgg at 48 h, control (A), montmorillonite (B), kaolinite (C), goethite (D).

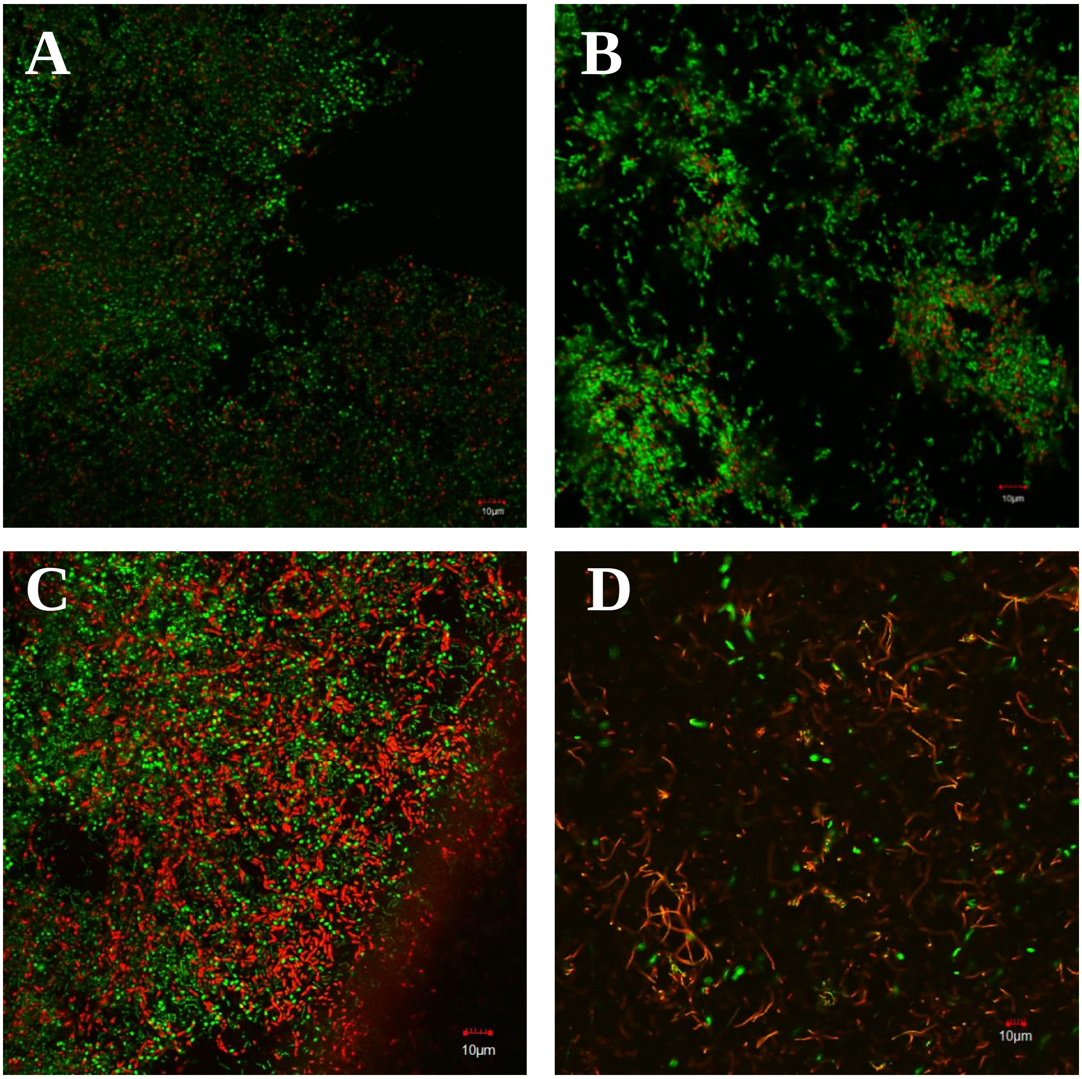

Figure S2. CLSM images of live/dead staining of *B. subtilis* cells after 24 h exposure to control (A), montmorillonite (B), kaolinite (C), goethite (D).
